# Supplementary material for: Highly Efficient Protoplast Isolation and Transient Expression System for Functional Characterization of Flowering Related Genes in Cymbidium Orchids
Source: Int J Mol Sci. 2020 Mar 25;21(7):2264. doi: 10.3390/ijms21072264 (PMC7177621; doi:10.3390/ijms21072264)
Supplement: Supplementary file 1 [file ijms-21-02264-s001.zip › ijms-746197-supplementary/Table S1.pdf]

Table S1. Primer sequences used in this study

	Primer name	Primer sequence（5′→3′）*
**Protein subcellular localization**	PAN580-CsAP3-F	CTTAAGTCCGGAGCTAGCTCTAGAGATGGGGAGAGGGAAGATAG
	PAN580-CsAP3-R	TCGCCCTTGCTCACCATGGATCCAGCGAGACGCAGATCATGAGG
	PAN580-CsPI-F	CTTAAGTCCGGAGCTAGCTCTAGAGATGGGACGTGGAAAGATAG
	PAN580-CsPI-R	TCGCCCTTGCTCACCATGGATCCCTTGTTTCCCTGCAAGTTG
**BiFC**	pSPYNE-CsAP3-F	TGGAGAGAACACGGGGGACTCTAGAATGGGGAGAGGGAAGATAGAG
	pSPYNE-CsAP3-R	GTCGACAGTACTATCGATGGATCCAGCGAGACGCAGATCATGAGG
	pSPYCE-CsPI-F	TGGAGAGAACACGGGGGACTCTAGAATGGGACGTGGAAAGATAG
	pSPYCE-CsPI-R	GTCGACAGTACTATCGATGGATCCCTTGTTTCCCTGCAAGTTG
**Transient expression**	O-FT-PAN-F	AGCTAGCTCTAGAGACGTCTCGAGGACCGGTCATGAATAGAGAGAGAGACTC
	O-FT-PAN-R	ATTGCCAAATGTTTGAACTGCAGCCGGGCGGCCGTCAATCCTGCATCCTTCTTCCGC
	O-SPV1-PAN-F	AGCTAGCTCTAGAGACGTCTCGAGGACCGGTCATGGCGAGGGAGAAGATACAGAT
	O-SVP1-PAN-R	ATTGCCAAATGTTTGAACTGCAGCCGGGCGGCCGTCACTTCCAACCAGCACATGA
**qRT-PCR**	Q-FT-F	GGCAGGAAGTGATGTGCTATG
	Q-FT-R	GCGAAGTCCCTGGTGTTGA
	Q-SVP-F	GCTTTCTATTCTTTGCGATGC
	Q-SVP-R	GGTTTGGATATGTTCCTTGACTG
	Q-SOC1-F	GGGAAGGACGGAGATGAGG
	Q-SOC1-R	ATCGCAGAGCACGGACAAC
	Q-AP1-F	CTGAGGTCGCTCTAATCGTG
	Q-AP1-R	GTGCCGTTTGCTCTTCTGT
	Q-LFY-F	TTGGGGATTTGGGGTTTAC
	Q-LFY-R	GATGATTGTGATGGTGGGAG
	CsUBQ-F	CCGGATCAGCAAAGGTTGA
	CsUBQ-R	AAGATTTGCATCCCTCCCC

* The sequences on the lines are the fusion sequence.
