# Supplementary figures and images for: Highly Efficient Protoplast Isolation and Transient Expression System for Functional Characterization of Flowering Related Genes in Cymbidium Orchids
Source: Int J Mol Sci. 2020 Mar 25;21(7):2264. doi: 10.3390/ijms21072264 (PMC7177621; doi:10.3390/ijms21072264)

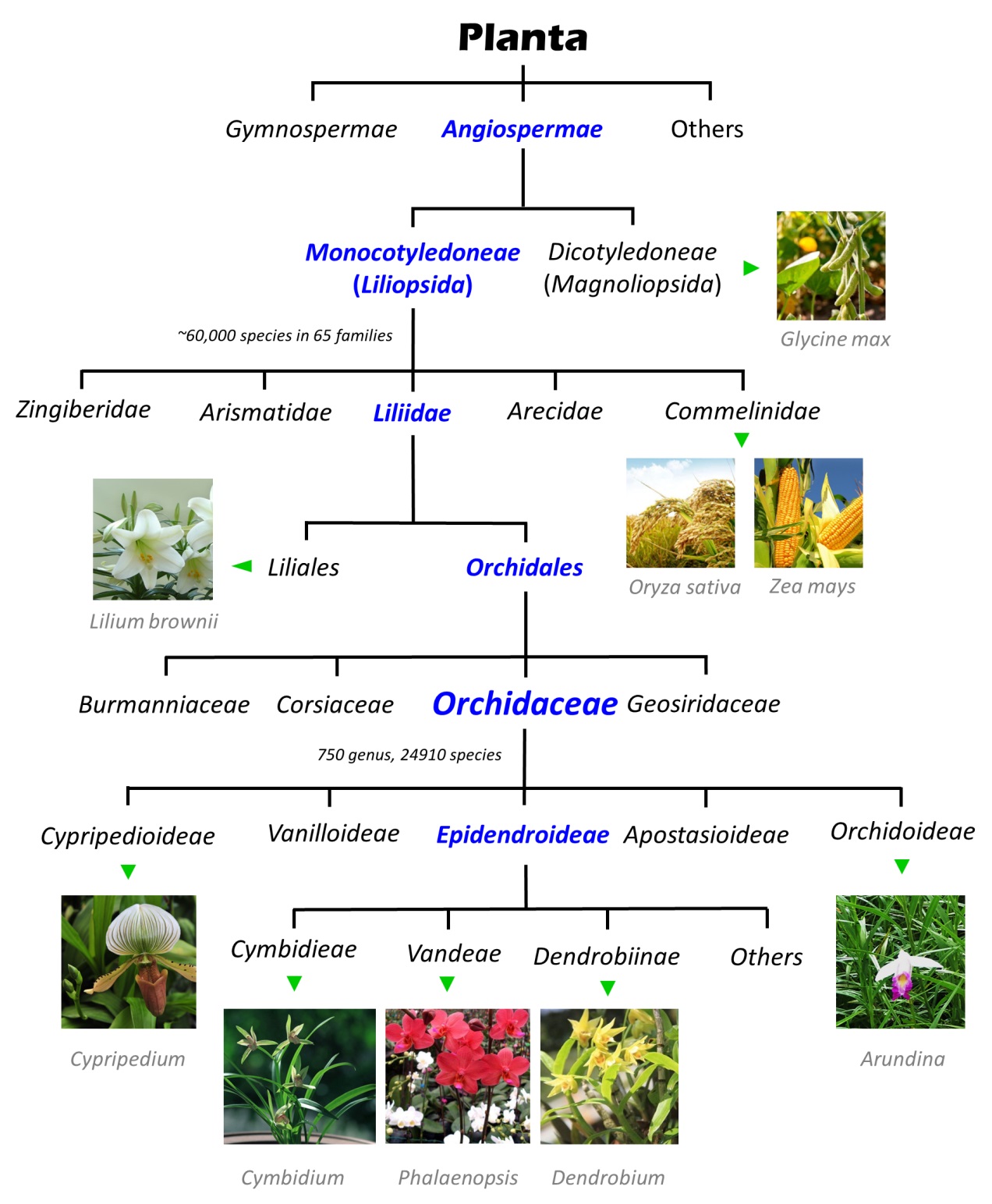

Supplement: Supplementary file 1 [file ijms-21-02264-s001.zip › ijms-746197-supplementary/Figure S1.jpg]

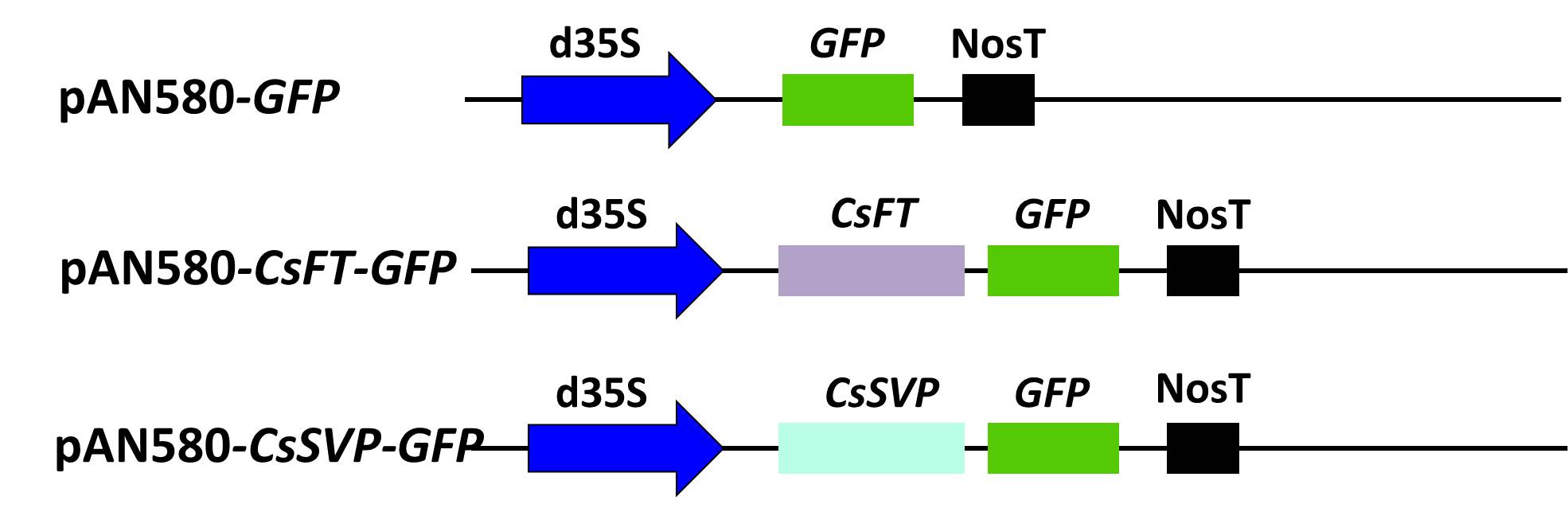

Supplement: Supplementary file 1 [file ijms-21-02264-s001.zip › ijms-746197-supplementary/Figure S2.jpg]
